# X-Ray Causes mRNA Transcripts Change to Enhance Orai2-Mediated Ca^2+^ Influx in Rat Brain Microvascular Endothelial Cells

**DOI:** 10.3389/fmolb.2021.646730

**Published:** 2021-09-14

**Authors:** Fangfang Xu, Yang Wang, Huiwen Gao, Xinchen Zhang, Yu Hu, Tingting Han, Bing Shen, Lesha Zhang, Qibing Wu

**Affiliations:** ^1^ School of Basic Medical Sciences, Anhui Medical University, Hefei, China; ^2^ Department of Radiotherapy, The First Affiliated Hospital of Anhui Medical University, Hefei, China; ^3^ Department of Otolaryngology-Head and Neck Surgery, Lu’an People’s Hospital, Lu’an Affiliated Hospital of Anhui Medical University, Lu’an, China

**Keywords:** x-ray, high-throughput sequencing, brain microvascular endothelial cell, Orai2, store-operated calcium entry

## Abstract

**Background:** Radiation-induced brain injury is a serious and treatment-limiting complication of brain radiation therapy. Although endothelial cell dysfunction plays a critical role in the development of this pathogenesis, the underlying molecular mechanisms remain elusive.

**Methods:** Primary cultured rat brain microvascular endothelial cells (BMECs) were divided into five groups without or with exposure of x-rays delivered at 5 Gy or 20 Gy. For the irradiated groups, cells were continued to cultivate for 12 or 24 h after being irradiated. Then the mRNA libraries of each group were established and applied for next-generation sequencing. Gene ontology and Kyoto Encyclopedia of Genes and Genomes pathway enrichment analyses were conducted to analyze the sequencing results. Quantitative polymerase chain reaction, western blotting, cck8 assay and intracellular calcium concentration assays were conducted to analyze the role of Orai2-associated SOCE in x-ray induced cellular injury.

**Results:** In total, 3,005 transcripts in all the four x-ray–exposed groups of BMECs showed expression level changes compared with controls. With the dose of x-ray augment and the following cultured time extension, the numbers of differentially expressed genes (DEGs) increased significantly in BMECs. Venn diagrams identified 40 DEGs common to all four exposure groups. Functional pathway enrichment analyses indicated that those 40 DEGs were enriched in the calcium signaling pathway. Among those 40 DEGs, mRNA and protein expression levels of Orai2 were significantly upregulated for 24 h. Similarly, calcium influx via store-operated calcium entry, which is modulated by Orai2, was also significantly increased for 24 h in x-ray–exposed BMECs. Moreover, the change in SOCE was suppressed by btp-2, which is a non-selective inhibitor of Orai. Additionally, x-ray exposure induced a significant decrease of proliferation in BMECs in the dose- and time-dependent manner.

**Conclusion:** These findings provide evidence for molecular mechanisms underlying BMECs dysfunction in development of radiation-induced brain injury and suggest new approaches for therapeutic targets.

## Introduction

Brain radiotherapy is a critical and effective treatment for intracranial tumors (Balentova and Adamkov, 2015). It has also long been recognized as primary management of nasopharyngeal carcinoma (Lee et al., 2019; Sun et al., 2019). However, radiation-induced brain injury remains a major common and serious complication of this therapy, with the severity of the injury determined by the radiation dose, irradiated brain volume, and accompanying treatment (Andrews et al., 2017; Wang et al., 2018). Patients with brain injury often experience impairments in memory, attention, cognitive function, and language skills or even death (Burns et al., 2016; Wang et al., 2019a). Thus, it is of the utmost importance to understand brain injury induced by radiotherapy to improve its therapeutic effect.

Radiation has a profound and progressive effect on the neurons, glia, and vasculature of the brain, causing molecular, cellular, and functional changes (Balentova and Adamkov, 2015). However, the molecular mechanisms underlying radiation-induced brain injury are not fully understood. According to previous studies, radiation may directly damage brain parenchymal cells or provoke damage to the vascular system, leading to brain ischemia (Yang et al., 2017). Emerging evidence suggests that radiation damages the vascular system by inducing endothelial cell apoptosis, reducing vessel density and length, increasing vascular permeability and vascular endothelial growth factor expression, and disrupting the blood-brain barrier (Li et al., 2004; Brown et al., 2005; Jin et al., 2014). Although radiation-induced brain injury is influenced by numerous and complicated factors, endothelial cell dysfunction plays a primary role in its development (Balentova and Adamkov, 2015; Tripathi et al., 2019). Therefore, it is crucial to elucidate the mechanisms underlying endothelial cell dysfunction to aid in the prevention and treatment of radiation-induced brain injury.

Proteomics and transcriptomics analyses have been applied in the study of long-term radiation-induced vascular dysfunction (Azimzadeh et al., 2015). However, such data assessing the molecular mechanisms of radiation-induced vascular dysfunction are insufficient and incomplete. A recent study by our team analyzed x-ray–treated (20 Gy) brain microvascular endothelial cells (BMECs) with next-generation sequencing and found that this radiation causes 383 genes to be significantly differentially expressed in BMECs and that these genetic alterations are accompanied by functional changes in the calcium (Ca^2+^) signaling pathway (Wu et al., 2020). In the present study, we used next-generation sequencing to further investigate the effects of two delivered doses of ionizing radiation (5 and 20 Gy) and of time after the exposure on transcriptional expression changes in BMECs.

Because ionizing irradiation induces alterations in the Ca^2+^ signaling pathway in BMECs (Wu et al., 2020), it is necessary to determine the molecular mechanisms underlying the potential Ca^2+^ signaling pathway–induced brain injury. The most widely known pathway for entry of Ca^2+^ into cells is through store-operated Ca^2+^ entry (SOCE), and SOCE is modulated by STIM, a Ca^2+^ sensor located in the endoplasmic reticulum, and by proteins in the Orai family, which form highly selective Ca^2+^ channels (Moccia et al., 2019). Therefore, Orai proteins and the associated SOCE may be involved in radiation-induced brain injury. Veath et al. have provided evidence that Orai2 modulates the magnitude of SOCE by forming heteromeric channels (Vaeth et al., 2017).

Given the plausibility for the involvement of Orai proteins and SOCE in radiation-induced brain injury and the finding by (Vaeth et al., 2017), a primary goal of the present study was to test the hypothesis that the levels of *Orai2* expression and the magnitude of SOCE are increased in BMECs exposed to x-rays. We used high-throughput sequencing, real-time quantitative polymerase chain reaction (qPCR), and intracellular Ca^2+^ concentration ([Ca^2+^]_i_) assays to assess whether this molecular mechanism may be related to BMEC dysfunction and contribute to radiation-induced brain injury.

## Materials and Methods

### Primary Culture of BMECs

All animal experiments were conducted in compliance with the National Institutes of Health (publication No. 8523). The Animal Experimentation Ethics Committee of Anhui Medical University (No. LLSC 20200179) reviewed and approved the study protocol and handling of the animals. Total of six Sprague Dawley rats (littermates, aged 2–3 weeks and weighing 10–20 g, sex in half) were purchased from the Animal Center of Anhui Medical University. The rats had free access to food and water and were house in a room with a 12-h light/dark cycle. The temperature of the room was maintained at 22–25°C. Tissue attainment from the rats for the harvest of BMECs and the primary BMECs dispersion were conducted as previously described (Zhang et al., 2007). Briefly, a gas mixture containing 100% oxygen and 2% isoflurane with the volume speed of 0.2 L/ min was used to anesthetize rats. Then the rats were humanely killed by inhalation of excess carbon dioxide (CO_2_) and cleansed three times with 75% alcohol. All subsequent procedures were conducted under aseptic conditions. The brain and its cerebral hemispheres were removed and placed in a petri dish containing a sterile phosphate-buffered saline solution. The pia mater, surface blood vessels, and cerebral medulla were removed from the cerebral cortex. The harvested tissue was cut into pieces, homogenized into suspensions, and filtered through an 80 µm mesh screen. The resulting filtrate was then filtered through a 200 µm mesh screen. The oversize residue was collected and centrifuged at 1,000 rpm for 5 min at 4°C. The resulting precipitate was collected and digested with 0.2% type II collagenase (Sigma-Aldrich, St. Louis, MO, United States) in phosphate-buffered saline at 37°C for 30 min and then centrifuged at 1,000 rpm for 5 min at 4°C. Finally, the obtained precipitate was resuspended in Endothelial Cell Medium containing 20% fetal bovine serum, 1% endothelial cell growth supplement, and 1% penicillin/streptomycin solution. The growth of other types of cells and impurities was reduced through the differential adhesion method, and the cell culture medium was replaced 4 h later. Cells were incubated at 37°C in 5% CO_2_. The medium was replaced every 3 days.

### BMECs X-Ray Exposure

A CLINIC600C linear accelerator (Varian, United States) was used to irradiate BMECs, at a rate of 2.5 Gy/ min. Cells cultured to 60% confluence were irradiated with 5 Gy or 20 Gy. The x-ray source was 100 cm from the cells and had an irradiation range of 20 cm × 20 cm. The cells in the control group were not irradiated by x-rays, but received all other treatments that the x-ray–exposed group received. The primary cultured BMECs were divided into five groups: (1) unexposed controls; (2) 12 h after delivery of 5 Gy of radiation; (3) 24 h after delivery of 5 Gy of radiation; (4) 12 h after delivery of 20 Gy of radiation; and (5) 24 h after delivery of 20 Gy of radiation. All experiments were conducted with three biological replicates.

### RNA Preparation

RNA was extracted using a commercially available kit (Vazyme Biotech Co., Ltd, Nanjing, China). BMECs with or without radiation exposure were digested and resuspended in lysis buffer RL1. The cell suspension was transferred to a genomic DNA filter column and centrifuged at 13,000 × *g* for 2 min. The resulting supernatant was collected and buffer RL2 was added. The mixture was placed in an RNAPure column and centrifuged again. The precipitate was collected. Buffer RW1 and buffer RW2 were used to rinse the RNAPure columns. The columns were put into new RNase-free collection tubes. RNAase-free ddH_2_O (60 μL) was added to the central part of the columns. The columns remained at room temperature for 2 min to dissolve RNA, and were then centrifuged at 13,000 × *g* for 1 min to elute RNA. A spectrophotometer was used to determine the purity and concentration of the extracted RNA. The optical density ratio of 260–280 nm was between 1.8 and 2.2, which met experimental requirements. Then messenger RNAs extractions were performed using Poly A selection of Illumina TruSeq RNA sample preparation kit (Illumina, San Diego, CA, United States) to establish the RNA sample libraries. In specific, the total RNAs were undergone two rounds of purification using poly-T oligo-attached magnetic beads. During the second elution of the poly-A RNA, the RNA was also fragmented.

### High-Throughput Sequencing and Data Processing

The high-throughput sequencing platform Illumina HiSeq 2,500 was used to analyze the RNA samples. The sequencing length was 150 base pairs. All the quantity and quality of RNA sample came up to the standards of high-throughput sequencing. FastQC software (Patel and Jain, 2012) was used to assess sequence quality, and Trimmomatic software (version 0.3) Bolger et al. (2014) was used to filter adaptors and lower mass readings. The quality-approved sequences were mapped to the rat reference genome (National Center for Biotechnology Information [NCBI] genome assembly, version Rnor_6.0, the genome assembly download site is https://www.ncbi.nlm.nih.gov/genome/73?genome_assembly_id=203777) with HISAT2 (version 2.0.13) (Kim et al., 2015), converted to a binary BAM file through samtools (version 1.9) (Li, 2011), and assembled into single complete transcripts through StringTie (version 1.3.5) software (Pertea et al., 2015). The transcripts were then annotated according to the National Center for Biotechnology Information’s annotation file (.gtf) Rnor 6.0. To quantify the expression level of each transcript, we utilized the most commonly used method, that is, fragments per kilobase being presented as per million fragments mapped. By using the blood-brain barrier RNA sequencing (RNA-Seq) quantification software Kallisto (Bray et al., 2016), the number of known mRNA sequences was counted. Transcript expression levels of samples from each of the five groups were analyzed to compare differences between each two groups by using edgR package software (http://bioconductor.org/packages/2.4/bioc/html/edgeR.html) (Robinson et al., 2010). A negative binomial model was used to calculate the significance of the gene expression level changes among the groups.

### Gene Ontology and Kyoto Encyclopedia of Genes and Genomes Pathway Enrichment Analyses

The differentially expressed transcriptomes (with a 1.5-fold change cutoff) from each of the four x-ray–exposed groups were obtained, and those that were found in all four groups were further analyzed by GO and KEGG pathway enrichment analyses and gene expression pattern analyses (Ashburner et al., 2000; Kanehisa and Goto, 2000). The Metascape database was used to analyze and annotate enrichment of the functional pathways for the proteins encoded by DEGs. GO annotation was conducted with the Metascape online tool. GO annotation was used to describe the functions of the screened DGEs in the three GO domains: molecular function, biological process, and cellular component. KEGG pathway analysis of the DEGs was also carried out in the same database. Significantly upregulated or downregulated genes based on the obtained RNA-Seq data were regarded as DEGs, and a two-sided *p* < 0.05 was considered statistically significant.

### Gene Expression Pattern Analysis

The expression patterns of the DEGs found in all four x-ray–exposed groups were further revealed using R package TCseq (version 1.10.0) (Wang et al., 2019b), focusing on those characteristics that changed with time after irradiation. Euclidean distance was used as a measure of the distance between samples. The fuzzy c-means clustering algorithm was applied to cluster genes having the same expression pattern over time.

### qPCR Analysis

The RNA reverse transcription reaction system (20 μL) contained RNA (1 μg), 50 μM Oligo (dT)_18_ Primer (1 μL), 50 ng/ μL random hexamers (1 μL), HiScript Enzyme Mix (2 μL) (Vazyme Biotech Co., Ltd, Nanjing, China), 2 × Reaction Mix (10 μL), and sufficient diethyl pyrocarbonate–treated water to bring the total volume to 20 μL. The reaction was performed using the following conditions: 25°C for 5 min, 50°C for 15 min, and 85°C for 5 min. The qPCR reaction for the collected cDNA was conducted using the Roche LightCycler 480 II PCR instrument (Basel, Switzerland) according to the SYBR Green I Master method. A mixture containing 2 × AceQ qPCR SYBR Green Master Mix, forward primer, reverse primer, cDNA, and ddH_2_O was used in the amplification reaction. The reaction was carried out in three stages: (1) denaturation at 95°C for 5 min; (2) 40 cycles of 95ºC for 10 s and 60ºC for 30 s; and (3) dissociation at 95 °C for 15 s, 60 ºC for 60 s, and 95°C for 15 s. A melt curve was used to analyze each reaction product. To detect possible carryover or contamination, negative controls were conducted for every sample. The mRNA levels were normalized against β-actin*.* The primer sequences for β*-*actin were 5′-CCC​ATC​TAT​GAG​GGT​TAC​GC-3′ (forward) and 5′-TTT​AAT​GTC​ACG​CAC​GAT​TTC-3′ (reverse). The primer sequences for *Orai2* were 5′-ACC​TTG​TGA​TTG​GCA​GGG​TA-3′ (forward) and 5′-ACT​GCG​ATA​TCC​CAC​TGG​AG-3′ (reverse). Relative gene expression levels of the control and the x-ray–exposed groups were calculated according to the 2^−ΔΔCT^ method.

### [Ca^2+^]_i_ Assay

[Ca^2+^]_i_ was measured using Fluo-8 AM as previously described (Guo et al., 2020). Briefly, cells were seeded on a cell culture cover glass and incubated in culture medium containing Fluo-8 AM (6 μM) and 0.02% pluronic F-127 for 30 min. Then the cells were mounted in a Ca^2+^ imaging chamber containing a Ca^2+^-free physiological saline solution with 140 mM NaCl, 2 mM MgCl_2_, 5 mM KCl, 0.2 mM EGTA, 10 mM glucose, and 10 mM HEPES brought to pH 7.4 with HCl or NaOH. To deplete intracellular Ca^2+^ stores, we added 4 μM thapsigargin and incubated the cells for at least 10 min. We added CaCl_2_ to initiate Ca^2+^ influx. N-(4-[3,5-bis(trifluoromethyl)-1H-pyrazol-1-yl]phenyl)-4-methyl-1,2,3-thiadiazole-5-carboxamide (btp-2, HY-100831, MedChemExpress) was used to block Orai channel and added to the solution. For analysis, the ratio of the real-time fluorescence intensity relative to the baseline fluorescence intensity (F_1_/F_0_) was used to represent the magnitude of the Ca^2+^ influx.

### Western Blotting

Equal amounts of protein were electrophoresed on 10% SDS-PAGE gels and transferred to PVDF membranes for immunoblotting. The membranes were blocked with a 5% non-fat milk dilution in TBST for 1 hour at RT and incubated with dilutions of primary antibody, which is Orai2 (20592-1-AP, Protein tech, dilution ratio of 1:1,000) or GAPDH (AF7021, Affinity, dilution ratio of 1:1,000) kept overnight at 4°C. After incubation with HRP-conjugated goat anti-rabbit IgG (E-AB-1003, Elabscience, dilution ratio of 1:5,000), chemiluminescence detection was performed by using the ECL plus Western blotting detection reagent (GE Health, Little Chalfont, United Kingdom), and immunoblots were quantified by densitometry using a chemiluminescence gel imaging analysis system (P and Q, Shanghai, China).

### BMECs Proliferation Assay

To detect the effect of x-ray on BMECs survival, the cell proliferation assay was performed as the manufactur’s instructions. The cells were coated on 96-well plates and irradiated with 5 Gy or 20 Gy. Then the cells were continue to cultivate for 12 or 24 h. The cells in the control group were not irradiated by x-ray. After that, cells were cultured with 10 μL cell counting kit-8 solution for 2 h at 37°C. To examine the optical density value, a microplate reader was used.

### Statistical Analysis

All data are presented as mean ± SEM. One-way analysis of variance, or *t* tests when only two groups were compared, was used to assess the data. Two-sided *p* values < 0.05 were considered statistically significant. GraphPad PRISM software version 8.3.0.538 (GraphPad Software, San Diego, CA, United States) was used for statistical analysis and for graphing.

## Results

### Quality of High-Throughput Sequencing

Five groups of cultured rat primary BMECs were sequenced. The raw data were trimmed and filtered using Trimmomatic software and evaluated *via* FastQC. All the raw data and differently expressed gene transcripts information are accessible via GEO accession numbers series GSE166073. GSE166073 study can be viewed at https://www.ncbi.nlm.nih.gov/geo/query/acc.cgi?acc=GSE166073. The median score of the reads was greater than 30, indicating that the quality of all samples was good (Supplementary Figure S1A).

The sequences that passed the quality check were further mapped and quantified via HISAT2 and StringTie software, using the rat reference genome and its annotation file in the Ensembl database (Cunningham et al., 2019). Each treatment group had three biological replicates. The mapping ratios of samples were above 90% in each group, indicating that the reference genome was suitable and that the sequencing samples were not contaminated (Supplementary Figures S1B–E).

To further evaluate the sequenced samples and to assess experimental reliability, we conducted correlation and principal component analyses of mRNA expression levels. Correlations were relatively high between the different biological replicates in each group (Supplementary Figures S2A–D). The results from the principal component analysis indicated clear heterogeneity in the RNA-Seq data between the irradiated cells and cells in the control group (Supplementary Figures S2E–H). Taken together, these results showed the sequencing data were reliable and of high quality.

### Identification of DEGs in BMECs

**FIGURE 1:**
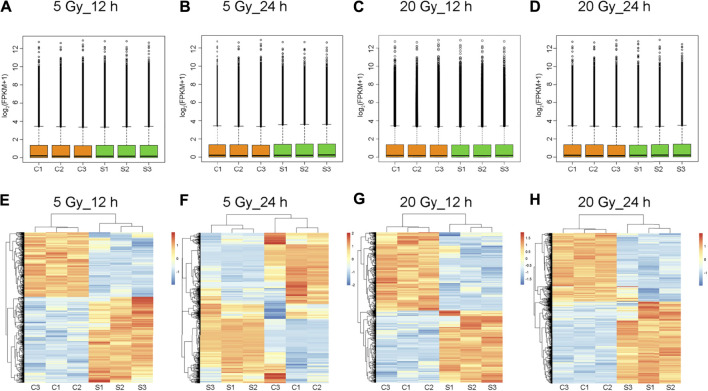
Total expression levels and cluster analysis of transcripts. **(A–D)** Overall transcript expression levels. C1, C2, and C3 represent control groups; S1, S2, and S3, x-ray–treated groups; FPKM, fragments per kilobase of exon model per million reads mapped. **(E–H)** Transcripts differentially expressed in brain microvascular endothelial cells exposed to the indicated x-ray dose compared with unexposed control cells; 12 h or 24 h is the time after x-ray exposure. Clustered heatmaps show combinations of transcripts with similar natures. Red indicates high expression level; blue, low expression. Log_2_ (FPKM + 1) values are scaled to different color depths.

**FIGURE 2:**
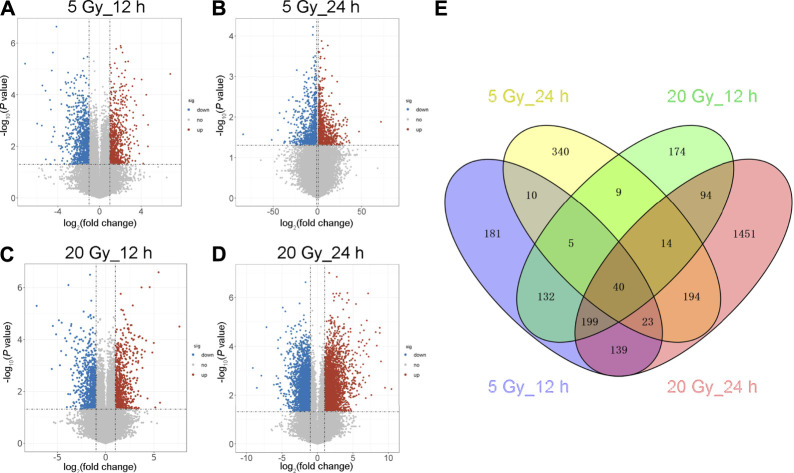
Volcano map and Venn diagram of differentially expressed genes (DEGs). **(A–D)** Volcano maps showing an overview of the differentially expressed transcripts (DEG cut points: corrected-*P* < 0.05 and absolute fold change ≥1.5) at the indicated x-ray dose (5 or 20 Gy) and time after exposure (12 or 24 h). Red dots represent significantly upregulated genes; blue dots, downregulated genes; gray dots, no significant change. **(E)** Venn diagram of the DEGs in the four irradiated groups. Blue shows 729 DEGs 12 h after 5 Gy; yellow, 635 DEGs 24 h after 5 Gy; green, 667 DEGs 12 h after 20 Gy; and red, 2,154 DEGs 24 h after 20 Gy. The intersection of the four groups has 40 DEGs.

Log_2_-transformed (FPKM + 1) values were used to evaluate the overall transcription expression levels in each group. No significant differences in overall expression levels were found between the control and treated groups (Figures 1A–D). The heat map of the cluster analysis (Figures 1E–H) indicated that compared with the control group, 12 h after exposure to a delivered x-ray dose of 5 Gy, the expression levels of 428 transcripts were significantly upregulated, whereas those of 301 transcripts were significantly downregulated; 24 h after exposure to the same dose, 375 transcripts were significantly upregulated, whereas 260 transcripts were significantly downregulated. In groups treated with a delivered x-ray dose of 20 Gy, 12 h after exposure, the expression levels of 452 transcripts were significantly upregulated and 215 transcripts were significantly downregulated; 24 h after exposure to this same dose, 1,052 transcripts were significantly upregulated and 1,102 transcripts were significantly downregulated. These findings indicated that the number of differentially expressed transcripts 24 h after 20 Gy was substantially higher than that 12 h after the exposure, suggesting that the change in transcript levels was time dependent. In addition, irradiation caused more DEGs 24 h after exposure to the higher dose (20 Gy) than after exposure to the lower dose (5 Gy). Volcano maps of the DEGs are shown in Figures 2A–D. To explore potential factors that similarly affected the development of the x-ray exposure–induced changes in transcript expression levels in BMECs, we used Venn diagrams to find 40 DEGs (Table 1) at the intersection of all four exposure groups (Figure 2E).

**TABLE 1:** Genes differentially expressed 12 and 24 h after x-ray exposure at doses of 5 and 20 Gy.

Gene name	Change	LogFC	*p* value
Ackr3	Upregulated	6.203854559	0.000120781
Aco2	Downregulated	0.366240402	0.001613528
Atp5pb	Downregulated	0.206566583	0.000110568
Bok	Downregulated	0.216086045	0.00430135
Calhm2	Upregulated	3.26635887	0.001206699
Ccdc117	Upregulated	16.05550716	0.000244579
Cebpd	Upregulated	2.193910563	0.011560722
Cyp2tl	Upregulated	2.839685804	0.035420269
Edn1	Upregulated	4.905550261	0.00000136
Egr2	Downregulated	0.181397059	0.000138533
Fas	Upregulated	6.10357644	0.000733553
Fos	Upregulated	14.47359244	0.000127806
Fzd7	Downregulated	0.02102981	0.000120406
Gdf15	Upregulated	32.35417257	0.000219431
Gdf6	Upregulated	7.364723843	0.000167684
Glrx3	Upregulated	2.390675075	0.000196573
Hint3	Upregulated	2.122373556	0.006211606
Hmox1	Upregulated	1.504562445	0.011803039
Kin	Upregulated	4.389965863	0.002010168
Klhl20	Upregulated	2.257815018	0.000152707
Lbh	Downregulated	0.13193082	0.0000252
Loc100912596	Upregulated	2.492649418	0.00193393
Lpl	Upregulated	4.092268918	0.000515649
Nr4al	Upregulated	18.07997089	0.001853707
Orai2	Upregulated	1.553667583	0.000913827
Pla2g16	Upregulated	1.660999051	0.019479562
Plk2	Upregulated	17.28180325	0.000104277
Psrc1	Upregulated	4.870876469	0.00805346
Puf60	Upregulated	2.290998899	0.034400671
Rab40c	Upregulated	6.213806244	0.003746853
Rac1	Downregulated	0.443506726	0.022679775
Rf00026	Downregulated	0.225225294	0.002679001
Rf00560	Downregulated	0.053448024	0.0000776
Sgk1	Downregulated	0.097961367	0.000252705
Svop	Upregulated	8.969642419	0.000216928
Tap1	Upregulated	5.102567172	0.010440122
Tfap2b	Upregulated	3.665482423	0.020941534
Tp53inpl	Upregulated	10.77674832	0.000839782
Zfp217	Upregulated	7.33061828	0.0000554
Zfp263	Up-regulated	6.579398825	1.44E-07

Note: LogFC represents the log of the fold change in the DEGs 24 h after exposure to x-rays at a dose of 20 Gy.

### Functional and KEGG Pathway Enrichment Analysis of DEGs

**FIGURE 3:**
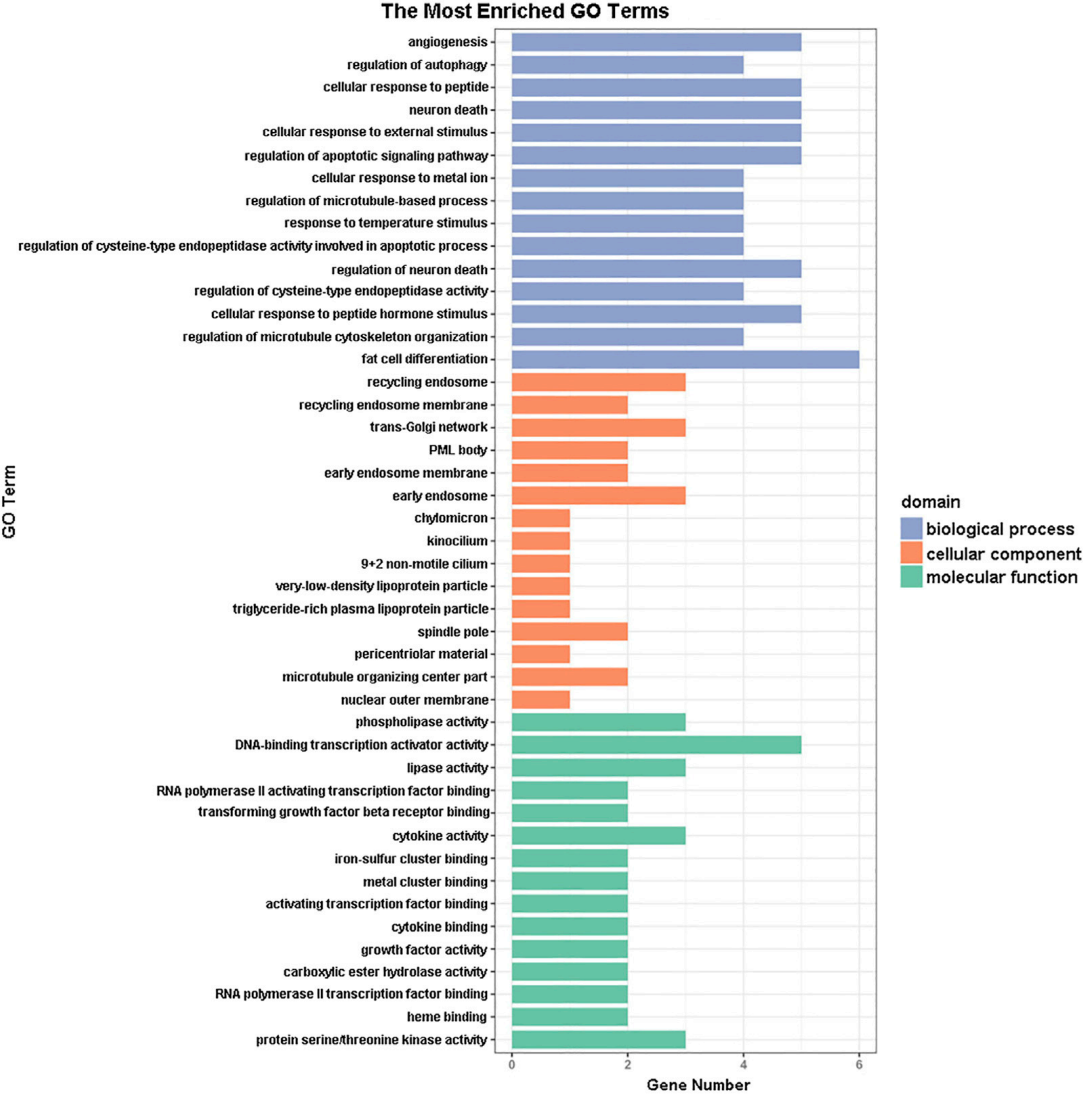
Gene ontology (GO) enrichment analysis results. The 15 most enriched GO terms in each of the three GO domains (molecular function, biological process, and cellular component) for the differentially expressed genes.

**FIGURE 4:**
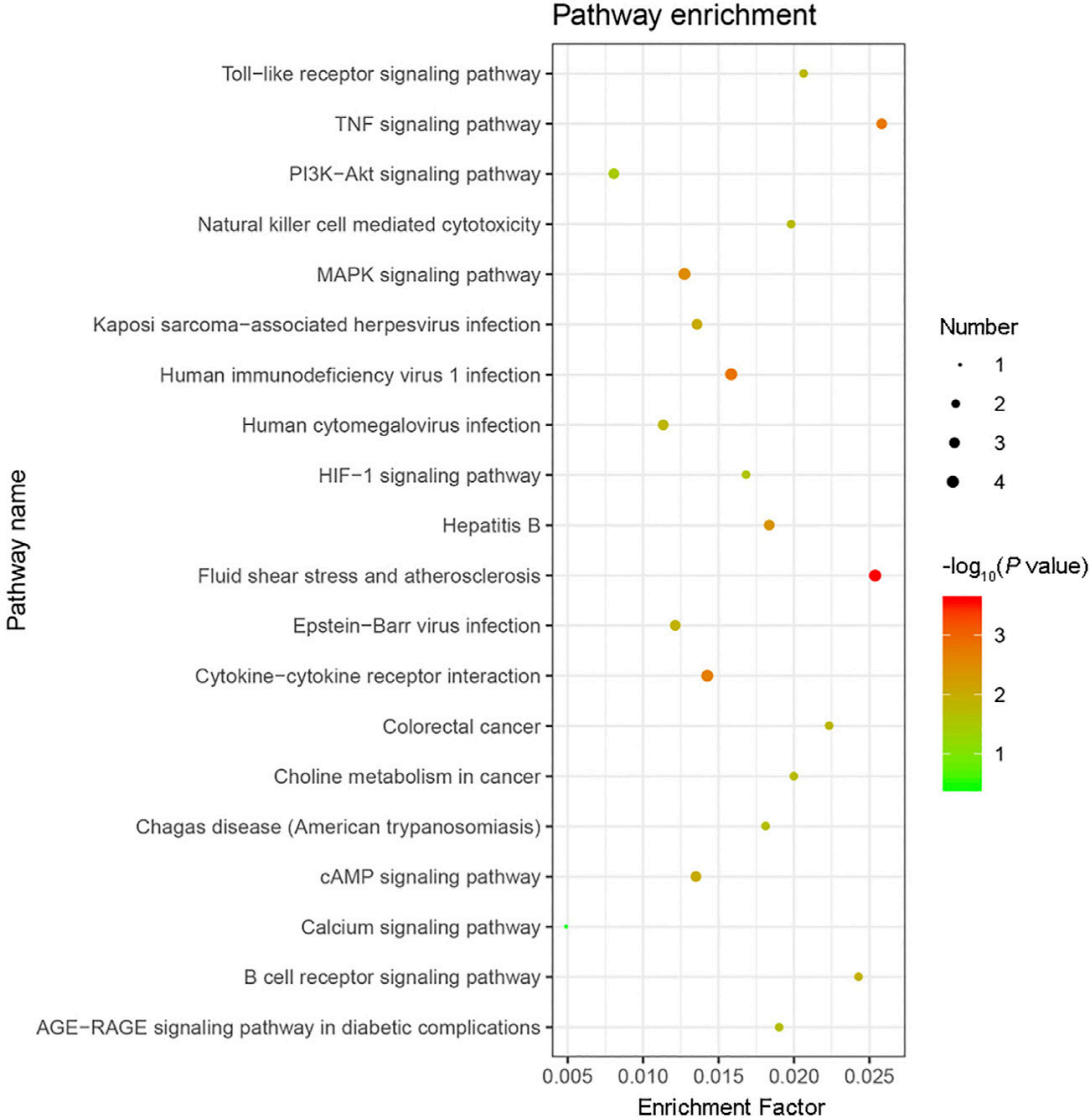
KEGG pathway enrichment analysis results. The top 20 KEGG pathways enriched with the 40 differentially expressed genes (DEGs) were analyzed by Metascape. The size of the circle indicates the number of DEGs enriched in the indicated pathway. Colors indicate P values as −log_10_(*P*), with red representing higher values, and green lower values (all P values are less than 0.05).

GO functional annotation of these 40 DEGs was performed using the Metascape online tool, and the molecular functions, biological processes, and cellular components enriched for these genes were assessed. The resulting enriched GO terms are shown in Figure 3. KEGG pathway analysis was conducted using the Metascape online tool, and the top 20 enriched pathways are given in Figure 4.

### Expression Pattern of DEGs

**FIGURE 5:**
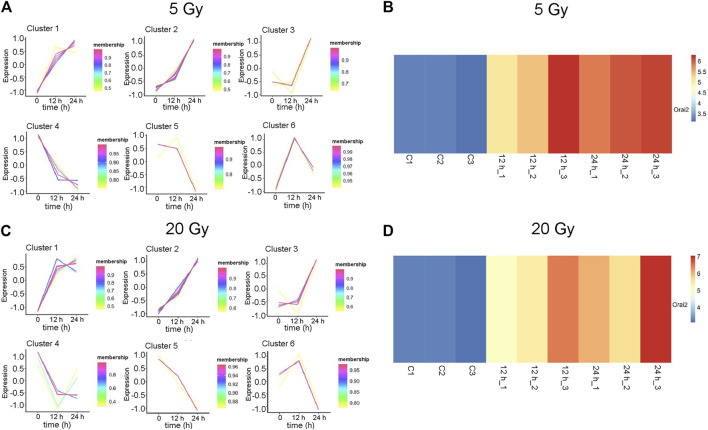
Expression patterns of 40 differentially expressed genes (DEGs). **(A–B)** Six gene expression pattern diagrams were drawn based on the expression levels of 40 DEGs 12 and 24 h after brain microvascular endothelial cells were irradiated with a delivered x-ray dose of **(A)** 5 Gy or **(B)** 20 Gy. **(C–D)** Overview of Orai2 expression profiles are shown as heatmaps for the respective control cells and 12 and 24 h after cells were exposed to 5 Gy **(C)** or 20 Gy **(D)**. Three biological replicates were used in each group. Blue and red indicate low and high expression, respectively.

The changes in the expression levels of the 40 DEGs with exposure time were created using TCseq software and are presented in Figures 5A,B. Six patterns were observed based on the change in expression levels from 1 to 12 h after x-ray exposure and from 13 to 24 h after exposure. Cluster one showed a pattern of continuous upregulation in transcript expression through 24 h, but the change in the slope of the upregulation during the second 12 h was shallower than that during the first 12 h. Cluster two also showed a pattern of continuous upregulation, but the change in the slope of the upregulation during the second 12 h was steeper than that during the first 12 h. Cluster three showed a slight downregulation in transcript expression during the first 12 h but a steep upregulated slope during the second 12 h. Cluster four showed a steep downregulation during the first 12 h but a stable or only slight downregulation in the second 12 h. Cluster five showed continuous transcript downregulation but the slope of the change during the second 12 h was steeper than that during the first 12 h. Cluster six showed a steep upregulation in transcript levels in the first 12 h followed by a steep downregulation in the second 12 h. The expression pattern of each of the 40 DEGs over 24 h following x-ray exposure is given by delivered radiation dose in Table 2.

**TABLE 2:** Differentially expressed gene level patterns over time in brain microvascular endothelial cells, by x-ray exposure dose.

Cluster pattern	Gene name	
	X-ray dose	
	**5 Gy**	**20 Gy**
1	*Orai2, Svop, Psrc1, Klhl20, Hint3, Glrx3, Gdf6, Edn1, Cyp2t1, Cebpd*	*Svop*, *Puf60*, *Pla2g16*, *Orai2*, *Klhl20*, *Hint3*, *Glrx3*, *Gdf6*, *Cebpd*
2	*Zfp263*, *Zfp217*, *Tp53inp1*, *Tap1*, *Rab40c*, *Plk2*, *Lpl*, *Loc100912596*, *Kin*, *Gdf15*, *Fas*, *Calhm2*, *Ackr3*	*Zfp263*, *Tp53inp1*, *Tap1*, *Rab40c*, *Psrc1*, *Loc100912596*, *Kin*, *Gdf15*, *Fas*, *Edn1*, *Cyp2t1*, *Calhm2*
3	*Nr4a1, Hmox1, Ccdc117, Fos*	*Rf00026*, *Rac1*, *Lbh*
4	*Tfap2b*, *Sgk1*, *Rf00560*, *Rac1*, *Fzd7*, *Egr2*, *Bok*	*Tfap2b*, *Fzd7*, *Egr2*, *Atp5pb*, *Aco2*
5	*Rf00026*, *Lbh*, *Aco2*	*Zfp217*, *Plk2*, *Nr4a1*, *Lpl*, *Hmox1*, *Fos*, *Ccdc117*, *Ackr3*
6	*Puf60*, *Pla2g16*, *Atp5pb*	*Sgk1*, *Rf00560*, *Bok*

The expression of *Orai2* appeared in Cluster pattern one for both radiation doses, indicating that expression levels of *Orai2* continued to increase for 24 h after exposure (Figures 5A,B; Table 2). The heat map analysis also showed that expression levels of *Orai2* increased in a time-dependent manner after irradiation (Figures 5C,D). Given that Orai proteins form Ca^2+^ release-activated Ca^2+^ channels and mainly regulate Ca^2+^ influx and the Ca^2+^signaling pathway via SOCE (Moccia et al., 2019), we hypothesized that x-ray exposure would increase *Orai2*-associated SOCE in BMECs over time.

### The Effect of X-Ray Treatment on Orai2-Associated SOCE in BMECs

**FIGURE 6:**
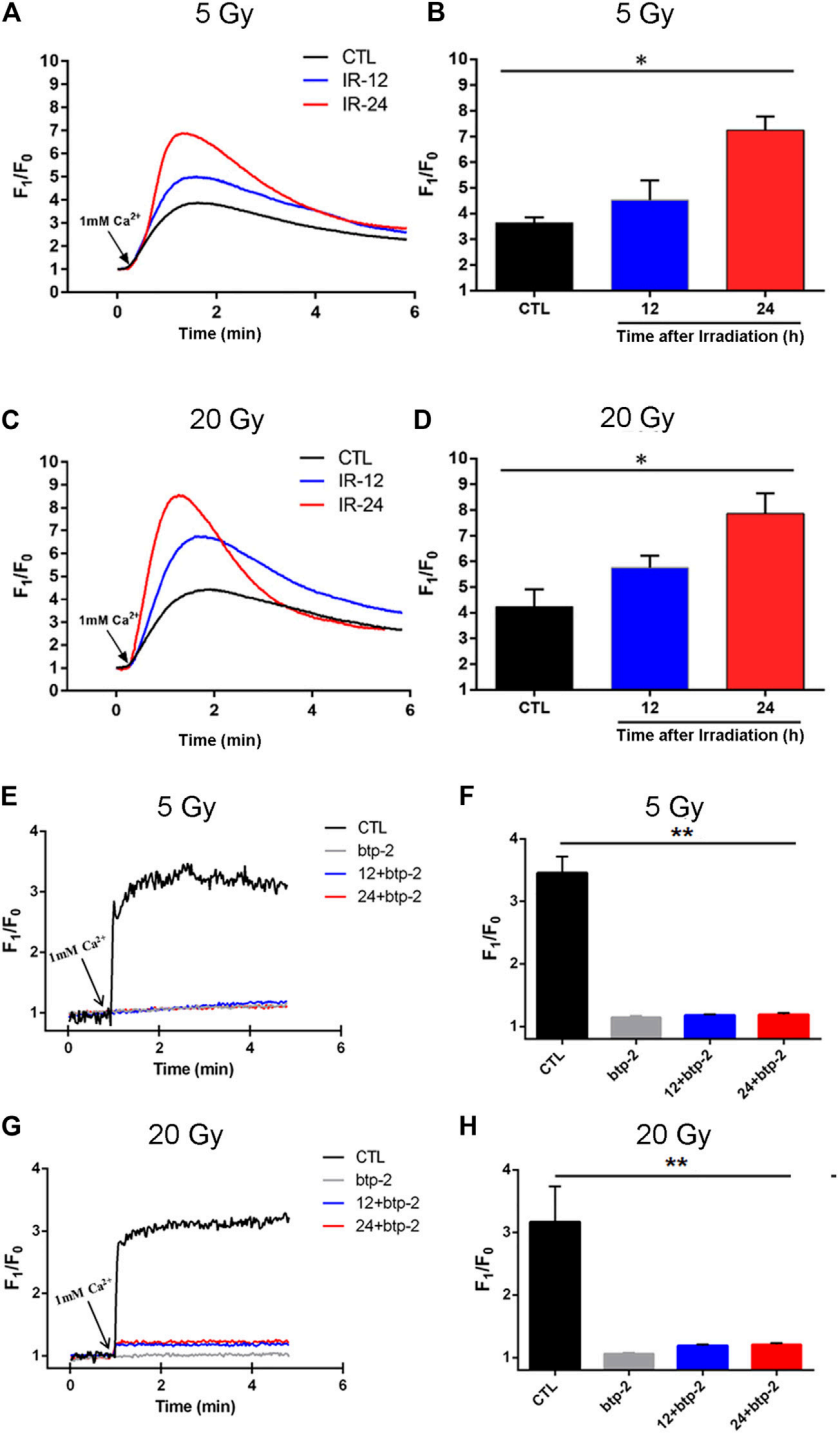
Effect of x-ray exposure on Orai2*-*associated store-operated Ca^2+^ entry (SOCE) in brain microvascular endothelial cells (BMECs). **(A–D)** Changes in the magnitude of Ca^2+^ entry via SOCE in the BMECs. Representative traces **(A, C)** and summary data **(B, D)** showing SOCE evoked by the addition of thapsigargin (2 µM) in BMECs 12 h (IR-12) or 24 h (IR-24) after the delivery of 5 Gy **(A, B)** or 20 Gy (C, D) of ionizing radiation or not (CTL). SOCE was achieved by re-introducing 1 mM Ca^2+^ to a Ca^2+^-free bath solution. F_1_/F_0_ represents the magnitude of the Ca^2+^ influx. **(E–H)** Orai blocking inhibited the enhancement of store-operated Ca^2+^ entry (SOCE) induced by x-ray exposure in BMECs. Representative traces **(E, G)** and summary data **(F, H)** showing thapsigargin-evoked SOCE in BMECs. 10 µM btp-2 was added to a Ca^2+^ -free bath solution. Data are shown as the mean ± SEM (*n* = 6). *p < 0.05, **p < 0.01 *vs.* the control group.

To test this hypothesis, BMECs mounted on cell culture cover glass were irradiated with delivered doses of 5 Gy or 20 Gy and cultured for 12 h or 24 h. The [Ca^2+^]_i_ was then determined. Compared with the control group, the magnitude of the Ca^2+^ influx into irradiated cells was significantly higher after the Ca^2+^stores were depleted (Figures 6A–D). Meanwhile, the magnitude of the Ca^2+^ influx through SOCE was significantly higher in cells cultured for 24 h vs. those cultured for 12 h after being irradiated with either 5 Gy or 20 Gy. Thus, SOCE appeared to be enhanced in x-ray–exposed BMECs in a time-dependent manner. Moreover, to elucidate the correlation between Orai and Ca^2+^ level in x-ray induced BMECs injury, btp-2 was used to non-selectively inhibit Orai. From Figures 6E–H, btp-2 significantly suppressed the increase of SOCE induced by x-ray exposure in BMECs. When compared with the CTL group, which presents calcium influx immediately increased after 1 mM Ca^2+^ was added, when 10 µM btp-2 was added to a Ca^2+^-free bath solution, enhancement of SOCE was blocked whether the BMECs were undergone either 5 Gy or 20 Gy of x-ray exposure. Overall, the above results present that *Orai*2-associated SOCE involves in the changing induced by irradiation in BMECs.

### X-ray Exposure Increases the Expression of Orai2 and Decreases the Proliferation Rates of BMECs.

**FIGURE 7:**
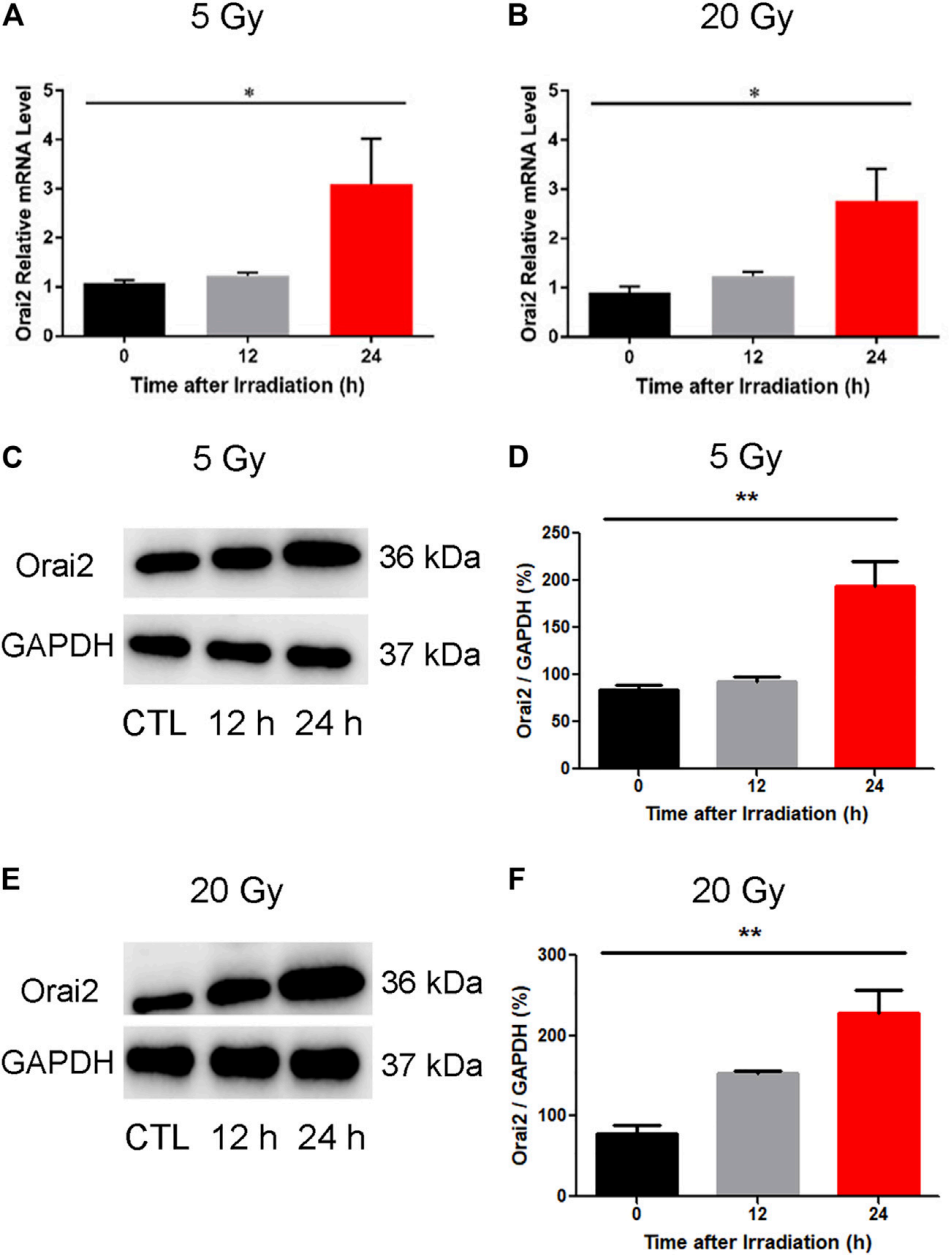
Effect of x-ray exposure on the expression of Orai2 on the mRNA and the protein levels. **(A–B)** Orai2 mRNA expression levels in irradiated BMECs relative to controls are significantly increased 24 h after 5 Gy **(A)** or 20 Gy **(B)**. Data are shown as the mean ± SEM (*n* = 3–6). *p < 0.05 *vs*. the control group. **(C–F)** Effect of x-ray exposure on Orai2 expression in brain microvascular endothelial cells. Representative images **(C, E)** and summary data **(D, F)** showing Orai2 expression in the cells exposed with 5 Gy or 20 Gy x-ray and continuing cultured for 12 h or 24 h. The control group were not irradiated. GAPDH was loading as a control. Data are shown as the mean ± SEM (*n* = 4). *p < 0.05, **p < 0.01 *vs*. the control group.

To confirm our earlier RNA-Seq findings indicating an upregulation of Orai2 expression levels following x-ray exposure, we used qPCR and western blotting. As shown in (Figures 7A,B), the mRNA expression levels of Orai2 significantly increased over time in BMECs receiving either dose, consistent with the RNA-Seq results. The protein levels of Orai2 were also significantly increased over time either after exposure of 5 Gy or 20 Gy x-ray, this trend was consistent with that of Orai2 on the mRNA level (Figures 7C–F). Additionally, when the cells were exposed with either 5 Gy or 20 Gy x-ray and continuing cultured for either 12 h or 24 h, the cell proliferation rates were lower than that of control group which was not treated by irradiation in the dose- and time-dependent manner (Supplementary Figure S3). This finding provides supporting evidence for an association between Orai2 and SOCE and cellular injury following x-ray exposure.

## Discussion

Although radiotherapy is used for treating primary intracranial tumors and metastases, the brain is still the major dose-limiting organ owing to the complications of radiation-induced brain injury (Balentova and Adamkov, 2015; Yang et al., 2017). Investigating the dose-dependent pathophysiological development of such brain injury is essential for the management of radiotherapy and the discovery of drug targets to relieve damage. Endothelial cell dysfunction plays a critical role in the pathogenesis of radiation-induced brain injury. Specifically, radiation induces endothelial cell apoptosis, increases vascular permeability, and disrupts the blood-brain barrier (Li et al., 2004; Brown et al., 2005; Jin et al., 2014). However, how the underlying mechanisms of endothelial dysfunction and endothelial function change over time and with dose of radiation remain unclear. We previously investigated the effect of a delivered x-ray dose of 20 Gy on transcript expression in BMECs by using high-throughput sequencing (Wu et al., 2020), a powerful tool for revealing changed genetic patterns and potential therapeutic targets of disease (Ma et al., 2010; Azorsa et al., 2016). The present study followed up on that work and further explored transcript expression alterations with two doses (5 Gy or 20 Gy) and times (12 h or 24 h) following x-ray exposure. High-throughput sequencing results indicated that the number of DEGs after 20 Gy was significantly increased 24 h after the exposure compared with 12 h after exposure at the same dose. The higher dose of radiation caused more DEGs than the lower dose 24 h after exposure. We found 40 DEGs common to both doses and times after exposure and grouped them into six expression changing patterns. GO and KEGG pathway enrichment analyses showed the pathways in which the 40 DEGs were involved. Our results from both RNA-Seq and qPCR analyses also indicated that *Orai2* was upregulated with exposure to either dose and remained upregulated for at least 24 h. Moreover, western blotting results showed that Orai2 expression levels were significantly increased over time either after exposure of 5 Gy or 20 Gy x-ray. Our [Ca^2+^]_i_ assay results suggested that x-ray exposure induced an enhancement of SOCE in a time-dependent manner, and the increase could be inhibited by btp-2, a non-selective inhibitor of Orai. This is, to our knowledge, the first report on how the dose and time following exposure to x-rays influence the genetic profile, including changes in mRNA *Orai2* expression, and alter SOCE in BMECs.

Many studies have previously shown that endothelial cell apoptosis is involved in radiation-induced brain injury (Peña et al., 2000; Li et al., 2004). Among the 40 DEGs determined by high-throughput sequencing in the present study, we found several that regulate cell apoptosis. Bok is a member of the Bcl-2 family, which is an important antiapoptotic factor in BMECs (Ueda et al., 2005). Previous studies have reported that Bcl-2 expression is significantly decreased in BMECs during oxygen glucose deprivation–induced injury and ischemia-reperfusion injury (Yang et al., 2012; Chen et al., 2018). Our sequencing results showed that Bok was downregulated in BMECs exposed to x-rays; such downregulation may promote endothelial cell apoptosis. By contrast, Fas, which encodes the Fas cell surface death receptor, was upregulated. Fas plays a key role in the positive regulation of programmed cell death. It has been reported that Fas mediated the apoptotic response of mouse testis cells to ionizing radiation (Embree-Ku et al., 2002). As shown in Table 2, the expression of Fas appeared in the “Cluster two″ pattern, which continued to increase for 24 h after x-ray exposure, for both the 5 Gy and the 20 Gy dose. These results provide supporting evidence that Fas upregulation may enhance cell apoptosis after radiation. However, further study is warranted to verify these changes and our speculation.

Irradiation induces an increase in brain vascular permeability (Yuan et al., 2003; Yuan et al., 2006). In the present study, we found changes in genes related to the regulation of vascular permeability, including atypical chemokine receptor 3 (*Ackr3*) and frizzled family receptor 7 (*Fzd7*). *Ackr3* encodes a member of the G protein–coupled receptor family. Ackr3 is considered a scavenger of the CXC chemokine CXCL12, which prevents leukocytes from entering the microvasculature of the brain; thus, upregulation of Ackr3 promotes blood-brain barrier impairment and inflammatory brain injury (Cruz-Orengo et al., 2011; Silwedel et al., 2018). Our results showed that *Ackr3* was upregulated in BMECs exposed to x-rays. By contrast, *Fzd7* was significantly downregulated. The expression pattern of *Fzd7* was in “Cluster 4,” which continues to decrease for a least 24 h after x-ray exposure, in both the 5 Gy and the 20 Gy group*. Fzd7* encodes frizzled7, a receptor for Wnt proteins. Inhibition or deletion of frizzled7 induces elevated permeability of cells and vessels by regulating the Wnt canonical signaling pathway (Ferreira Tojais et al., 2014). Thus, our findings provide evidence supporting the importance of *Fzd7* in the development of radiation-induced brain injury. We speculate that the changes in *Ackr3* and *Fzd7* together cause an increase in endothelial permeability. However, further experimental study will be needed to confirm these changes and potential effects.

A series of prior studies has shown that Ca^2+^ influx or even the accumulation of Ca^2+^ significantly increases vascular permeability and cell apoptosis (Jho et al., 2005; Pinton et al., 2008; Bates, 2010). Radiation induces an increase in [Ca^2+^]_i_, with a dose-dependent increase in some types of cells (Decrock et al., 1864; Liu et al.eng, 2017; Bo et al., 2020). Among the many factors that modulate Ca^2+^influx, Orai2 is an important protein, playing a key role in SOCE (Scremin et al., 2020). Our results showed that *Orai2* is upregulated in a time-dependent manner, increasing 12 h after x-ray exposure and increasing further after 24 h, consistent with the observed time-dependent increase in SOCE. It has been reported that the expression level of Orai2 is significantly increased in pulmonary arterial smooth muscle cells from patients with idiopathic pulmonary arterial hypertension (Fernandez et al., 2015). Enhanced expression of the *Orai2* transcript has also been found in endothelial cells of lung explants obtained from patients with idiopathic pulmonary arterial hypertension (Saygin et al., 2020). In addition, overexpression of Orai2 is responsible for enhanced SOCE in endothelial progenitor cells and pulmonary artery smooth muscle cells, whereas silencing Orai2 significantly attenuates thapsigargin-induced SOCE in the acute myeloid leukemia cell line HL60 (Diez-Bello et al., 1864; Fernandez et al., 2015; Dragoni et al., 2014; Chen et al., 2017). On the basis of such previous study results, we speculate that the upregulation of *Orai2* contributes to the increase in the magnitude of Ca^2+^ entry via SOCE, leading to increased [Ca^2+^]_i_. To test this hypothesis, we used btp-2 to block Orai2 activity. Our results indicated that the enhancement of SOCE induced by x-ray exposure were significantly inhibited by btp-2. Therefore, Orai2 was responsible for the change in SOCE. As to the injury of BMECs induced by the irradiation, we observed the effect of x-ray exposure on proliferation of BMECs by cell counting kit-8 assay (Supplementary Figure S3). When the cells were exposed with either 5 Gy or 20 Gy x-ray and continuing cultured for either 12 h or 24 h, the cell proliferation rates were lower than that of control group which was not treated by irradiation in the dose- and time-dependent manner. Therefore, the result shows that x-ray injury could directly inhibit the growth of BMECs. Moreover, according to the functional and KEGG pathway enrichment analysis of DEGs from the sequencing data, we can also indicate the changed gene transcripts primarily enrich in the biological pathways relating to regulation of autophagy, neuron death, apoptotic process. The above evidence all could support the x-ray induced injury of BMECs. Evidences indicate that intracellular Ca^2+^ signaling is critical to promote the endothelial repair, among which SOCE most probably therapeutically induce the regrowth of wounded vessels by spur endothelial progenitor cells homing (Moccia et al., 2014). These imply that Orai2-mediated Ca^2+^ influx enhancement somehow may take part in the developmental injury of BMECs induced by x-ray.

In addition to the upregulation observed in the expression of the *Orai2* transcript, the Ca^2+^ signaling pathway appeared in our results of the KEGG pathway enrichment analysis for the 40 DEGs common to all x-ray–exposed groups in the present study. Besides the Ca^2+^ signaling pathway, the results of the top 20 pathways enriched for these 40 DEGs also included the MAPK signaling pathway, PI3K-Akt signaling pathway, and HIF-1 signaling pathway. Previous studies have shown that radiation induces MAPK pathway activation, which in turn causes inflammatory and apoptotic responses (Nagaraja and Nagarajan, 2018; Mantawy et al., 2019). Rutin protected against radiation-induced neurotoxicity via regulation of PI3K-Akt signaling pathway (Thabet and Moustafa, 2018). Mounting evidence has shown that HIF-1 inhibition enhances the effect of radiotherapy and decreases the development of radiation-induced necrosis (Meng et al., 2018; Yang et al., 2018). In addition, results from our GO functional enrichment analysis indicated that the 40 DEGs were enriched, among other relevant processes, in the regulation of apoptosis, angiogenesis, and neuron death.

## Conclusion

In summary, our findings showed that x-ray exposure induced changes in levels of transcript expression in BMECs, with alterations in some transcripts dose- or time-dependent. We found that the 40 DEGs common to the two doses and times following exposure were enriched in the regulation of the Ca^2+^signaling pathway, apoptosis, and angiogenesis as well as other processes. Among these DEGs, *Orai2* may play a pivotal role in radiation-induced brain injury. We speculate that the observed upregulation in the level of Orai2 expression and the observed enhancement in SOCE lead to intracellular Ca^2+^ accumulation, which eventually contributes to radiation-induced brain injury. These results provide evidence for a potential molecular mechanism underlying radiation-induced brain injury and may shed new light on approaches to mitigate or prevent such injury.

## Data Availability

The data presented in the study are deposited in the GEO repository, accession number GSE166073.
