# Supplementary figures and images for: X-Ray Causes mRNA Transcripts Change to Enhance Orai2-Mediated Ca2+ Influx in Rat Brain Microvascular Endothelial Cells
Source: Front Mol Biosci. 2021 Sep 14;8:646730. doi: 10.3389/fmolb.2021.646730 (PMC8477418; doi:10.3389/fmolb.2021.646730)

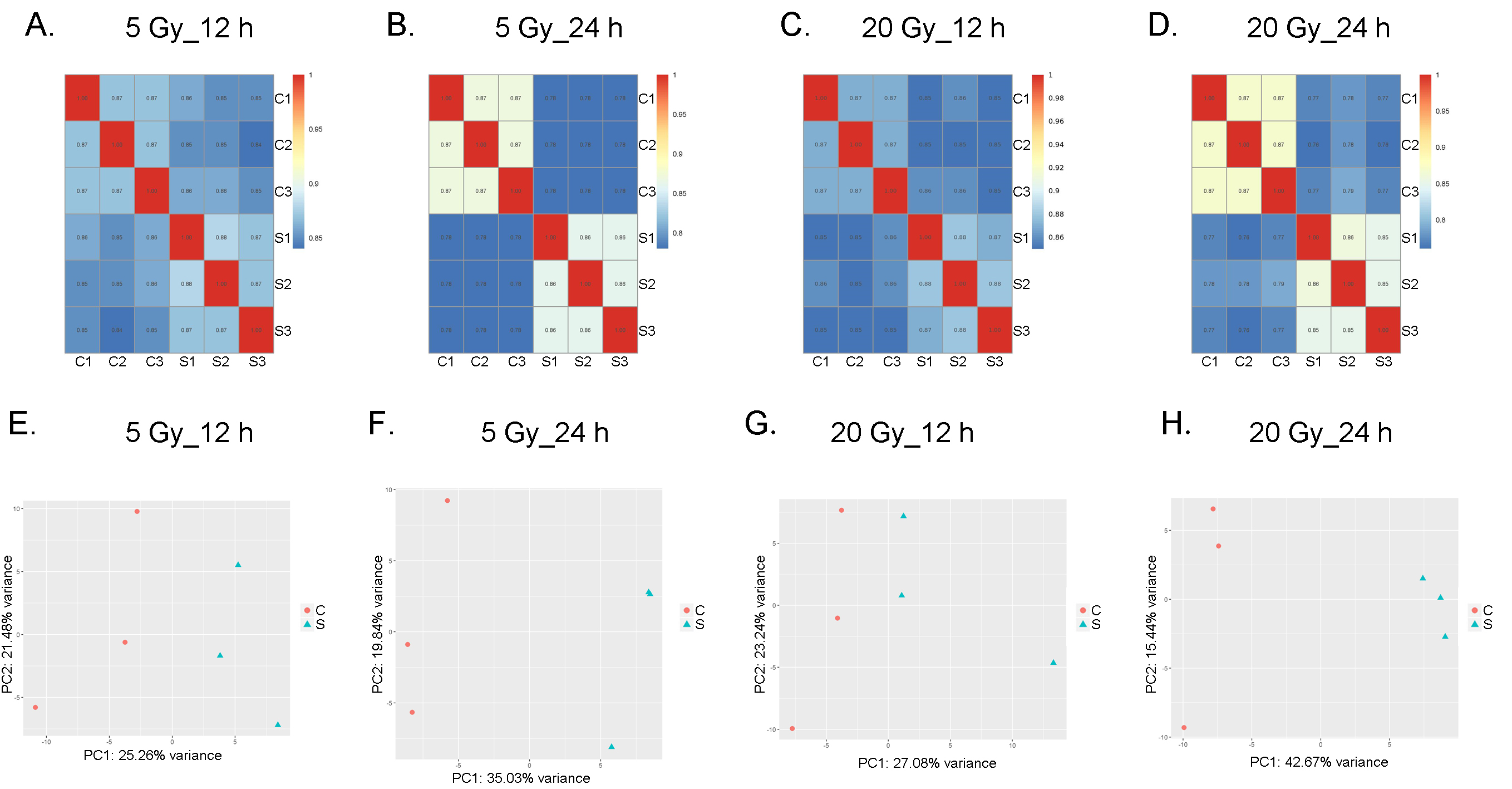

Supplement: Supplementary file 2 [file image2.tif]

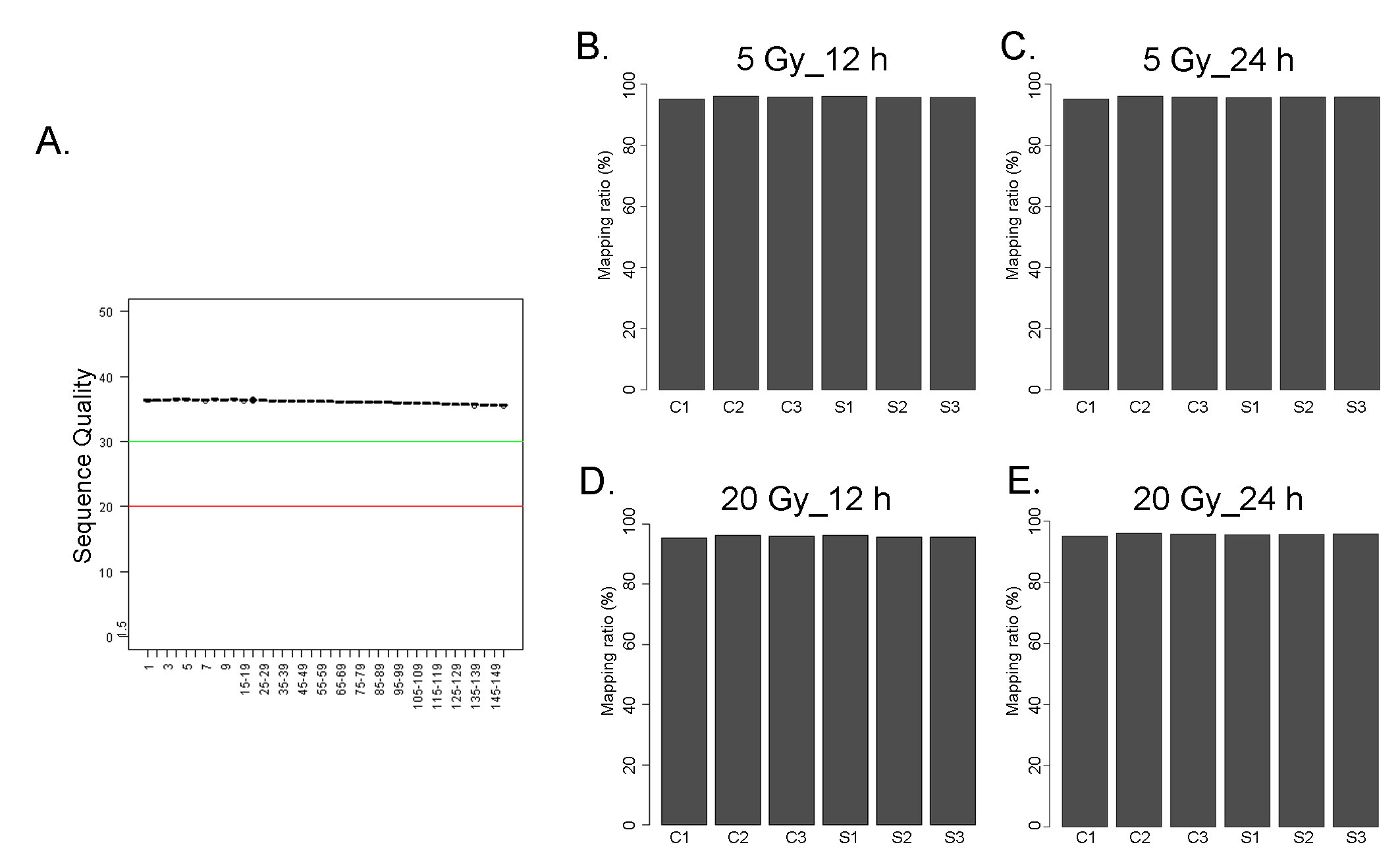

Supplement: Supplementary file 3 [file image1.tif]
